# Impact of RSUME Actions on Biomolecular Modifications in Physio-Pathological Processes

**DOI:** 10.3389/fendo.2022.864780

**Published:** 2022-04-21

**Authors:** Mariana Fuertes, Belén Elguero, David Gonilski-Pacin, Florencia Herbstein, Josefina Rosmino, Nicolas Ciancio del Giudice, Manuel Fiz, Lara Falcucci, Eduardo Arzt

**Affiliations:** ^1^ Instituto de Investigación en Biomedicina de Buenos Aires (IBioBA) - Consejo Nacional de Investigaciones Científicas y Técnicas (CONICET) - Partner Institute of the Max Planck Society, Buenos Aires, Argentina; ^2^ Departamento de Fisiología y Biología Molecular y Celular, Facultad de Ciencias Exactas y Naturales, Universidad de Buenos Aires, Buenos Aires, Argentina

**Keywords:** RSUME, RWDD3, SUMOylation, ubiquitin, pituitary, RCC, VHL, PTTG

## Abstract

The small RWD domain-containing protein called RSUME or RWDD3 was cloned from pituitary tumor cells with increasing tumorigenic and angiogenic proficiency. RSUME expression is induced under hypoxia or heat shock and is upregulated, at several pathophysiological stages, in tissues like pituitary, kidney, heart, pancreas, or adrenal gland. To date, several factors with essential roles in endocrine-related cancer appear to be modulated by RWDD3. RSUME regulates, through its post-translational (PTM) modification, pituitary tumor transforming gene (PTTG) protein stability in pituitary tumors. Interestingly, in these tumors, another PTM, the regulation of EGFR levels by USP8, plays a pathogenic role. Furthermore, RSUME suppresses ubiquitin conjugation to hypoxia-inducible factor (HIF) by blocking VHL E3-ubiquitin ligase activity, contributing to the development of von Hippel-Lindau disease. RSUME enhances protein SUMOylation of specific targets involved in inflammation such as IkB and the glucocorticoid receptor. For many of its actions, RSUME associates with regulatory proteins of ubiquitin and SUMO cascades, such as the E2-SUMO conjugase Ubc9 or the E3 ubiquitin ligase VHL. New evidence about RSUME involvement in inflammatory and hypoxic conditions, such as cardiac tissue response to ischemia and neuropathic pain, and its role in several developmental processes, is discussed as well. Given the modulation of PTMs by RSUME in neuroendocrine tumors, we focus on its interactors and its mode of action. Insights into functional implications and molecular mechanisms of RSUME action on biomolecular modifications of key factors of pituitary adenomas and renal cell carcinoma provide renewed information about new targets to treat these pathologies.

## Introduction

RSUME is a small protein containing an RWD domain that has a role in enhancing SUMO conjugation (1). The function of this domain is unknown and extends from amino acid 7 to 114 of the human protein. RSUME was first identified following a screen of GH3 pituitary tumor cells over-expressing gp130, which typically generate aggressive and highly vascularized tumors in nude mice (2).

In endocrine-related cancers, RSUME appears as responsible for regulatory actions over several factors with essential roles in tumorigenesis. For many of these effects, RSUME acts by modulating post-translational modification (PTM) of proteins. Given the strong connection between RSUME and PTMs, it is important to better understand how RSUME associates with regulatory proteins of ubiquitin and ubiquitin-like protein cascades and its pathophysiological consequences.

We briefly review the body of related work that is available on this field and discuss the mechanisms of action and regulatory impact of RSUME action. In none of the known actions and examples provided does the mechanism involve a modification on RSUME, but on the interacting protein, by modifying either its PTM or the interacting capability of this protein.

## RSUME Overview

**Figure 1:**
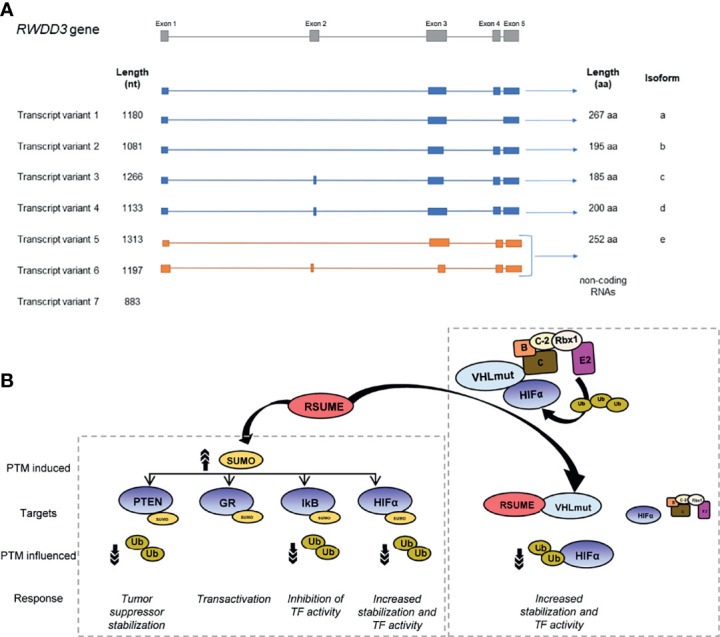
Scheme of human *RWDD3* transcript variants and RSUME targeted proteins. **(A)** Seven transcript variants of *RWDD3* human gene, of which five are translated into protein and two of these proteins are the best characterized proteoforms, RSUME-195 and RSUME-267. **(B)** RSUME interacts and enhances SUMOylation of targeted proteins (PTEN, GR, IkB and HIFα), affecting other PTMs, occurring in the same protein. The reduction of ubiquitination enhances activity of transcription factors (TFs) such as HIFα or transcription factor regulators (IkB, PTEN). RSUME promotes HIFα accumulation and activity by another mechanism independent of SUMOylation: the interaction of RSUME with VHL (the HIFα ubiquitin E3 ligase that promotes its degradation in normoxia) decreases VHL–HIFα binding and consequently HIFα ubiquitination.

Human RSUME is codified by the *RWDD3* gene located on chromosome 1. This gene generates seven mRNA splice variants, five of which code for different RSUME proteoforms (between 185 and 267 amino acids) and the rest are non-coding RNAs that are degraded by non-sense-mediated RNA decay due to their premature termination codons (3) (**Figure 1A**). RSUME is highly conserved in vertebrates (1) and is distributed in both the cytoplasm and the nucleus (1). *Rwdd3* gene gives rise to two mRNA splice variants in mouse and only one mRNA in rat, coding for two murine RSUME proteoforms (267 and 339 amino acids) and one rat RSUME protein (267 amino acids), respectively.

All RSUME proteoforms contain an RWD domain, a protein–protein interaction motif, which has been shown to share significant structural homology to the mammalian E2 SUMO-conjugating enzyme Ubc9 and the yeast E2 ubiquitin conjugase Mms2 (4). A comparative analysis showed that the core structure of RWD appears to be common among RWD-containing proteins, while the specificity of interaction and/or the function of each RWD-containing protein seems to be given by variations on the surface residues. This also suggests that each RWD domain interacts with different E2-conjugating enzymes in the same way (4).

Under stress conditions such as hypoxia, CoCl_2_ (hypoxia-mimicking stimulus), and heat shock, RSUME amounts were induced (1, 5) with a marked localization in the necrotic inner zone of tumor explants (1). Phosphorylation site prediction with KinasePhos software indicated that the RSUME protein sequence contains at least two putative JAK–STAT-sensitive tyrosine phosphorylation sites at positions 123 and 169 (6), a signaling pathway whose activation, either through elevated cytokine signaling during inflammation, or during hypoxic conditions, could lead to increased RSUME expression or activity (6).

The RSUME proteoforms are equally induced by hypoxia and exert similar actions, which may be related to the fact that all of them contain the same RWD domain (3). Since studies performed so far and revised here have been performed with the proteoform of 195 amino acids of length, the evaluation of others would be interesting in the future.

**Figure 2:**
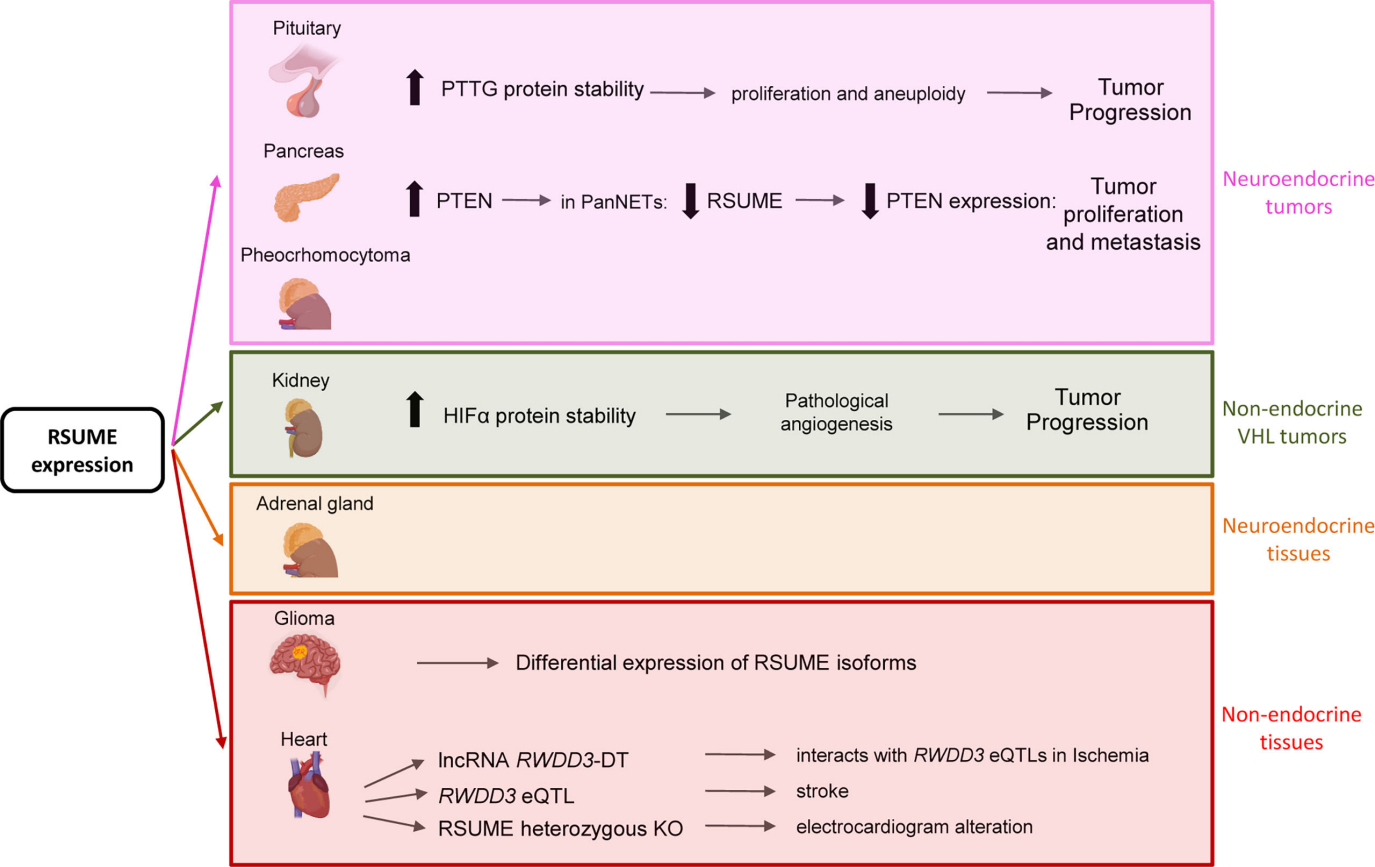
Summary of RSUME actions in neuroendocrine or non-endocrine tissues and tumors. RSUME is expressed and acts in normal and tumoral tissues. Of particular interest are those tissues in which it is highly expressed, such as pituitary, pancreas, kidney, brain and heart, where it exerts different functions through the interaction with the indicated key factors. Interestingly it is also expressed at high levels in normal and tumoral (pheochromocytoma) adrenal gland, a VHL type of tumor, in which its functions remain to be studied.

Tissue distribution of RSUME mRNA showed higher expression in cerebellum, pituitary, heart, kidney, liver, stomach, pancreas, adrenal gland, prostate, and spleen (1) (**Figure 2**). RSUME expression is upregulated in pituitary adenomas at mRNA (7) and protein levels (8, 9).

In glioblastoma tumors, RSUME is overexpressed and correlates with shorter overall survival time of patients (10). Mechanistically, RSUME downregulation in glioblastoma cancer cells leads to diminished proliferative and invasive abilities by modulation of the PI3K/AKT signaling (10). It is important to note that the distribution of RSUME proteoforms differs between glioma samples, demonstrating that while the shorter RSUME proteoform is present in all gliomas studied, the longest RSUME proteoform is differentially expressed in those samples (3). These results suggest separate roles for RSUME proteoforms in this kind of tumor, which remains an open question to be studied.

The *RWDD3* gene, between a subset of 16 genes, has been associated with breast cancer recurrence, metastases, and mortality in survival analyses in patients (11). Furthermore, genome-wide association studies (GWAS) in breast cancer patients showed an allele dose dependent association of single-nucleotide polymorphisms (SNPs) residing in *RWDD3* gene with time to neuropathy (common toxicity criteria), in patients undergoing taxane therapy (12).

In pancreatic neuroendocrine tumors (PaNETs), the down-modulation of RSUME expression is associated to an increased tumoral size and metastatic capacity in a murine model. Accordingly, PanNET patient´s tissues show reduced RSUME expression compared with normal pancreatic tissue. This action is mediated by its role in SUMOylation and stabilization of the tumor suppressor Phosphatase and Tensin Homolog deleted on Chromosome 10 (PTEN) (13) (**Figure 1B**).

## RSUME Contributes to VHL Syndrome Through Its Interaction With VHL and HIF

RSUME emerges as a tumor-associated protein since it is expressed in many other tumors such as pheochromocytoma, hemangioblastoma and renal carcinoma (RCC) (**Figure 2**). This leads to link RSUME to the von Hippel–Lindau (VHL) tumor syndrome (14), being those neoplasms the most frequently associated with the disease. VHL tumor syndrome is caused by mutations in *VHL* tumor suppressor gene. VHL-related tumors are highly angiogenic, originated by hypoxia-inducible factor-alpha (HIFα) deregulation (15). The mechanism mediated by RSUME to promote angiogenesis by HIFα stabilization has been described in clear cell RCC (16) and is discussed in detail below.

A bioinformatic analysis revealed that RSUME expression is increased in RCC tumors bearing VHL mutated form and it rises from earlier to late tumor stages during RCC progression (16). In this kind of cancer, high RSUME expression correlates with worse overall survival compared with patients expressing low RSUME levels (16). Metabolomics analysis unveiled that RSUME is also involved in metabolic changes associated to RCC malignant phenotype, particularly linked to fatty acid metabolism and antioxidant response (17). Thus, RSUME participates in tumor progression through several pathways.

## RSUME Expression in Pathological Tissue Responses

Besides its role in tumor progression, recent studies reveal that RSUME is connected with cardiac tissue response to ischemia and stroke (18, 19) (**Figure 2**). Enhanced SUMOylation has been proposed to protect from stroke and ischemia of the brain (20, 21). In line with this, RSUME has been associated with cardioembolic stroke in an Indian stroke patient´s study (19). Interestingly, in this GWAS study, the authors discovered loci related to different stroke types and located close to certain genes, where *RWDD3* is one of them. Further analysis revealed that this locus is an expression quantitative trait locus (eQTL) for RSUME and also associated with platelet distribution width and lipid metabolism (19), suggesting that RSUME expression levels are tightly regulated in stroke. This finding points RSUME level as an important factor to the proper response of cardiac tissue.

Another pathological response involving differential modulation of RSUME levels is neuropathic pain. Rojewska et al. found thirty-nine genes modulated by this pathology, of which RSUME together with six other genes are differentially repressed under sciatic nerve injury in a rat model and rescued after treatment that alleviates pain (22). This treatment has a key effect in reduction of the microglia activation and neuroinflammation. In fact, RSUME is expressed in brain but it shows higher expression in glial cells (https://www.proteinatlas.org/ENSG00000122481-RWDD3/single+cell+type) from human tissues. Further studies will be needed to unveil the role of SUMOylation, described as a regulator of brain development (23), and RSUME in normal or activated glia and pain.

## RSUME Regulates PTMs and Impacts in Different Cellular Processes

Since its discovery, RSUME has been linked to several regulators of PTMs. Through its RWD domain, RSUME presents structural similarity to UEVs (ubiquitin E2 variant proteins) and to E2 conjugases (4) that allow its interplay with ubiquitin and ubiquitin-like protein machinery. RSUME interacts with the E2-conjugating enzyme of the SUMO pathway, Ubc9, increases its activity, and colocalizes with it (1). RSUME also interacts with SUMO-1 (1).

Additionally, RSUME interacts physically with VHL protein (14), an essential component of the E3 ubiquitin ligase ECV complex, which also includes Elongin B, Elongin C, Cullin-2, and Rbx-1 (24) (**Figure 1B**). The most known targets of ECV E3 ubiquitin ligase are HIF-1 and 2α (25). VHL-RSUME interaction is independent of VHL-substrate, HIF-1α, as is demonstrated by *in vitro* interaction of these proteins. This observation is also confirmed by RSUME and VHL interaction in a cellular system with a mutant HIF unable to bind VHL (14). In spite of this, RSUME also interacts with VHL in the presence of the VHL–HIFα complex. Even more, increasing RSUME protein quantities displace HIF–VHL binding progressively (14), suggesting a competitive interaction between both proteins for VHL. This reduction in VHL–HIF binding suppresses HIFα ubiquitin conjugation by VHL promoting HIF stabilization. Additionally, RSUME interacts and downregulates the assembly of other components from the ECV complex (14, 16). Under hypoxia, VHL is SUMOylated by SUMO-1 conjugation predominantly at 171 lysine residue (K171) promoted by Protein Inhibitor of Activated STATγ (PIASγ) SUMO ligase (26). This SUMO-1 modification of VHL blocks its ubiquitin E3 ligase action on HIFα proteolysis (26). RSUME also increases VHL K171 SUMOylation (14). SUMO conjugation to VHL as well as RSUME-VHL interaction can suppress HIF-2α degradation, as demonstrated by HIF-2α stabilization mediated by RSUME in cells bearing VHL K171R mutation (16). In fact, RSUME is able to promote SUMOylation of VHL mutated variants observed in VHL syndrome, but also abrogates HIF-2α degradation by interacting with these VHL variants in a VHL-SUMOylation independent way (16). This HIF-2α accumulation has a major consequence in the context of RCC tumors, where angiogenesis becomes relevant for tumor growth, mainly promoted through the HIF-2α–VEGF pathway (27). In this cancer type, mutations in VHL gene are key drivers of carcinogenesis. As mentioned above, RSUME high expression is related to adverse prognosis in RCC patients. Mechanistically, RSUME promotes the wrong functioning of VHL mutated forms on HIF-2α degradation (16). Consequently, reduced angiogenesis is observed in RCC models carrying VHL mutations when RSUME is silenced (16). This RSUME action on HIF-2α ubiquitination mediated by VHL also occurs independent of VHL SUMOylation. It could be possible that both types of regulation, SUMOylation and protein interactions, act at different instances of VHL ubiquitin ligase activity.

RSUME also modulates two central players in inflammation. RSUME inhibits NF-kB activity through the stabilization of IκB (**Figure 1B**), which leads to the inhibition of two of its targets, interleukin-8 (IL-8) and cyclooxigenase-2 (Cox-2) (1). RSUME increases IκB SUMOylation at lysines 21 and 22, and enhances IκB protein stability in mammalian cells. RSUME also regulates the glucocorticoid receptor (GR) PTM (5) as detailed below. Further level of complexity appears because members of the PIAS family of SUMO E3 ligases also regulate GR-directed transcription (28) and HIF-1α SUMOylation (29), just like RSUME.

## Biomolecular Modifications by RSUME in Neuroendocrine Cells

The hypothalamus–pituitary–adrenal axis (HPA axis) is a complex neuroendocrine system in which the GR plays a central role in regulating inflammation, glucose and lipid metabolism, stress response, and development, among other important processes. Some PTMs such as phosphorylation, ubiquitination, and SUMOylation modulate GR activity (30–33). RSUME is an important regulator of heat shock-induced GR SUMOylation, by interacting with the GR and increasing its SUMOylation (5) (**Figure 1B**). The lysine K721 of GR is critical for the RSUME effect, showing that this site has a positive action on GR transcriptional activity and the expression of its endogenous target genes, FKBP51 and S100P. In addition, both K721 mutation and RSUME knockdown compromise coactivator GRIP1-mediated GR activation. Thus, RSUME manages GR-mediated transcription, modulating the cellular outcome to glucocorticoid exposure.

RSUME is a novel and important player in pituitary tumor pathogenesis. RSUME also acts on the HPA axis at the pituitary stage, increasing pituitary tumor transforming gene (PTTG) protein stability (9) (**Figure 1B**). PTTG is the vertebrate securin (34, 35) whose overexpression correlates with tumor invasiveness and recurrence. RSUME and PTTG are both upregulated in human pituitary adenomas (9, 36), and this positive correlation of expressions involves not only an increment of PTTG protein in pituitary tumor cells, but also an improved half-life of PTTG protein and a co-regulation with estrogens of the PTTG induction. Accordingly, RSUME upturns PTTG transcription factor (over targets such as c-Myc or cyclin D3) and securin activities, allowing the appearance in the tumor of aneuploid cells or multinucleated as a consequence. RSUME knockdown reduces securin PTTG and its tumorigenic potential in xenografted mice. This explains the effect of RSUME modulating PTTG high protein levels that account for PTTG tumor abundance and demonstrates an important role of RSUME in tumor cells of the pituitary. Regarding the molecular mechanism, we have described that PTTG protein levels decrease when the SUMOylation pathway is inhibited by the viral Gam1 protein (37), and the consequent reversal of this effect when an inactive mutant is used (9), suggesting that SUMO signaling is involved in the stabilization of PTTG. For many proteins, SUMOylation could protect them from degradation by the ubiquitin/proteasome system, in addition to increasing their stability, changing their subcellular localization or distribution, and/or modifying their molecular interactions. Furthermore, PTTG is targeted by other PTMs such as ubiquitination (38) or phosphorylation (39), which could also be modulated by RSUME.

In addition, RSUME is involved in pituitary adenoma progression by means of initiating pituitary tumor neovascularization through regulating HIF-1α levels and subsequent VEGF-A production under hypoxia in murine pituitary tumor cell lines and human pituitary adenoma cells (7, 8).

Regulation of protein stability by PTMs appears to be a key pathway in the control of these types of tumors. Almost half of ACTH-secreting pituitary tumors were reported to develop because of ubiquitin-specific peptidase 8 (USP8) somatic mutation (40), which leads to an increased USP8 deubiquitinating activity and triggers the release of adrenocorticotropic hormone (ACTH). Mutant USP8 inhibits EGFR ubiquitination and rescues it from proteasomal degradation, increasing the EGFR in the plasma membrane and returning to the cell surface by reversing the endocytosis and thus ultimately promoting ACTH secretion by activated EGFR signaling pathway (41). The overexpressed EGFR and E2F transcription factor 1 (E2F1) were implicated in the aggressiveness of pituitary tumors (42). E2F1 is also deubiquitinated and stabilized by a deubiquitinating enzyme named POH1 (43). Thus, the deubiquitinating enzyme USP8 could represent a great link within EGFR, E2F1, and ACTH in pituitary cancer.

## RSUME as a Biomodulator in Development

In an extensive transcriptomic study in 12.5- to 16-day-old rat embryos, *Rwdd3* gene was identified as part of a large set of upregulated genes of ganglionic eminence (GE), an embryonic structure that supplies the brain with diverse sets of GABAergic neurons (44). This pool of GE-enriched genes, including *Hod, Rwdd3, Nr2f2, Egr3, Cpta1, Cyp26b1*, and *Slit3*, may be important in telencephalic neural development. Taking into consideration the relevant role of SUMOylation in different regulatory mechanisms of brain development (23), future studies in *Rwdd3* KO mice will clarify its contribution in developmental processes in the brain.

RSUME has increasingly shown to be involved in cardiac pathologies. Ward et al. carried out a detailed study in which the comparison of ventricular tissue after and before ischemia in humans shows that several long non-coding RNAs (lncRNAs) are differentially expressed and related to fast response to ventricular ischemia (18). From the novel lncRNA group, a particular lncRNA targets five regulatory loci (expression quantitative trait loci, eQTL) for RSUME, and consequently, this lncRNA’s expression correlates with RSUME expression in this study group (18).

Similarly, the previously described GWAS study that associates an SNP affecting an RSUME’s QTL with cardioembolic stroke (19) provides a novel view for the hypoxia-mediated regulation of RSUME expression in cardiac tissue. Moreover, a different study found a suggestive locus near the RSUME gene that is involved in inter-individual levels of the Proprotein convertase subtilisin/kexin type 9 (PCSK9), which is a regulator of LDL receptor degradation and is associated with cardiovascular risk (45). Although the incidence of congenital heart defects is high, only a reduced number of them are caused by Mendelian inheritance (46). Noteworthy, defects in SUMOylation balance seem to play an important role in heart development as demonstrated by congenital heart defects observed in knockout mice for components of the SUMOylation system (46). Considering RSUME’s role in SUMOylation, it is likely that RSUME may modulate cardiac development, a hypothesis supported by the cardiac phenotypic alteration in electrocardiogram of heterozygous *Rwdd3* KO mice developed under an international project to discover new functions of genes [International Mouse Phenotyping Consortium (IMPC), www.mousephenotype.org]. Subsequently, RSUME gene has been included between essential genes, since its KO mice displayed homozygous lethality (47), which opens new interesting avenues in the study of *Rwdd3*.

## Conclusions and Future Perspectives

In the light of reported research at protein and mRNA levels, RSUME shows up as an important regulator of cellular function in various physiological and pathological processes, in which it appears as a promising biomarker and therapeutic target.

Although RSUME-mediated PTM regulation studies have been mainly performed in cancer, mainly neuroendocrine tumors, its participation in other processes including neuropathic pain and, more recently reported, stroke and ischemia has been described (**Figure 2**).

Since cancer must face an evolving environment, reversible modifications on proteins show additional regulation, more related to levels and cross-talk of PTMs in a specific moment. In this scenario, RSUME has an extensive participation by modulating key pathways such as VHL/HIF, PTTG, or NFkB.

Interestingly, two important mechanisms controlling pituitary growth, the regulation of PTTG protein stability and tumor abundance by RSUME, and the USP8/EGFR regulation, point to the involvement of the SUMO/ubiquitin pathways in pituitary pathogenesis.

VHL disease shows a non-predictable pattern of tumor development. The remaining questions about why some tissues are sensitive to tumor growth are still unanswered. In RCC, RSUME emerges as a modulator of VHL ubiquitin action on the HIF pathway, opening new perspectives on therapeutic strategies for this cancer type.

Considering the role of RSUME on regulatory mechanisms in several pathways, mostly described in pathology but relevant in physiology, research on RSUME KO will help to answer central questions about RSUME/RWDD3 modulatory actions, highlighting the relevance of coordinated PTMs.

## Author Contributions

MarF, BE, and EA conducted literature review, and conceptualized and wrote the manuscript. DG-P, FH, JR, NC-G, ManF, and LF conducted literature review, designed the figures, and contributed to editing the manuscript. All authors contributed to the article and approved the submitted version.

## Funding

This work was supported by the Max Planck Society from Germany (grant number 2012/2022); University of Buenos Aires (UBA) from Argentina (grant number N° 20020170100230BA); the Consejo Nacional de Investigaciones Científicas y Técnicas (CONICET) from Argentina (PUE-2016 N° 22920160100010CO); the Agencia Nacional de Promoción Científica y Tecnológica (ANPCyT) from Argentina (grant numbers PICT2014-3634, PICT2016-1620, and PICT-2018- 03232); and Fondo para la Convergencia Estructural de Mercosur (FOCEM) (grant number COF 03/11).

## Conflict of Interest

The authors declare that the research was conducted in the absence of any commercial or financial relationships that could be construed as a potential conflict of interest.

## Publisher’s Note

All claims expressed in this article are solely those of the authors and do not necessarily represent those of their affiliated organizations, or those of the publisher, the editors and the reviewers. Any product that may be evaluated in this article, or claim that may be made by its manufacturer, is not guaranteed or endorsed by the publisher.
